# Molecular evolution of the hyperthermophilic archaea of the Pyrococcus genus: analysis of adaptation to different environmental conditions

**DOI:** 10.1186/1471-2164-10-639

**Published:** 2009-12-30

**Authors:** Konstantin V Gunbin, Dmitry A Afonnikov, Nikolay A Kolchanov

**Affiliations:** 1 Institute of Cytology and Genetics of the Siberian Branch of the Russian Academy of Sciences, Novosibirsk, 630090, Russia; 2 Novosibirsk State University, Novosibirsk, 630090, Russia

## Abstract

**Background:**

Prokaryotic microorganisms are able to survive and proliferate in severe environmental conditions. The increasing number of complete sequences of prokaryotic genomes has provided the basis for studying the molecular mechanisms of their adaptation at the genomic level. We apply here a computer-based approach to compare the genomes and proteomes from *P. furiosus, P. horikoshii*, and *P. abyssi *to identify features of their molecular evolution related to adaptation strategy to diverse environmental conditions.

**Results:**

Phylogenetic analysis of rRNA genes from 26 Pyrococcus strains suggested that the divergence of *P. furiosus, P. horikoshii *and *P. abyssi *might have occurred from ancestral deep-sea organisms. It was demonstrated that the function of genes that have been subject to positive Darwinian selection is closely related to abiotic and biotic conditions to which archaea managed to become adapted. Divergence of the *P. furiosus *archaea might have been due to loss of some genes involved in cell motility or signal transduction, and/or to evolution under positive selection of the genes for translation machinery. In the course of *P. horikoshii *divergence, positive selection was found to operate mainly on the transcription machinery; divergence of *P. abyssi *was related with positive selection for the genes mainly involved in inorganic ion transport. Analysis of radical amino acid replacement rate in evolving *P. furiosus, P. horikoshii *and *P. abyssi *showed that the fixation rate was higher for radical substitutions relative to the volume of amino acid side-chain.

**Conclusions:**

The current results give due credit to the important role of hydrostatic pressure as a cause of variability in the *P. furiosus, P. horikoshii *and *P. abyssi *genomes evolving in different habitats. Nevertheless, adaptation to pressure does not appear to be the sole factor ensuring adaptation to environment. For example, at the stage of the divergence of *P. horikoshii *and *P. abyssi*, an essential evolutionary role may be assigned to changes in the trophic chain, namely, acquisition of a consumer status at a high (*P. horikoshii*) or low level (*P. abyssi*).

## Background

It is remarkable how the prokaryotes manage to survive and proliferate in the habitats exposed to an enormous spectrum of conditions despite their simplest organization. It is becoming increasingly apparent that the deep seas are the sources of novel microbial communities and of their so far unclear adaptive abilities. Microbial diversity is not only an academic issue, "it is a treasure house of innovation for the biotechnology industries" [1]. In the postgenomic era, comparative genomics keeps providing powerful tools for unraveling the molecular mechanisms of microbial adaptation. Complete genomic sequences of the prokaryotes give valuable information about the composition of genes, their function, arrangement order in the genome, operon structure, about single nucleotide substitutions in the coding and noncoding parts of the gene as well. High throughputs would allow to uncover the yet unclear molecular mechanisms of the adaptation of prokaryotes to challenging and/or novel environments at the level of genomic organization and protein structure [2].

Here we present the results of comparisons of the genomic sequences of the archaea Pyrococcus performed to elucidate the possible mechanisms of archaeal adaptation to life under different abiotic and biotic environmental conditions. Complete genome sequences are now available for three species of the Pyrococcus genus: *P. furiosus *[3], *P. horikoshii *[4], and *P. abyssi *[5]. The archaea of the genera Pyrococcus belong to the Thermococcales order [6,7]; those of the Thermococcales order are hyperthermophiles, strictly anaerobes and obligate heterotrophs [6,7]. Specific features of the *P. furiosus, P. horikoshii*, and *P. abyssi *habitats, certain differences between their genomes are summarized in Table 1. The optimal conditions of temperature, salinity, and pH are similar for their growth. However, their requirements for hydrostatic pressure and habitat depth are different despite the fact that they can proliferate also under pressures close to atmospheric [8]. *P. furiosus *can exist under pressures not above 20 MPa, it inhabits shallow hydrothermal vents. *P. horikoshii *inhabits at the depth of about 1400 m (~14 MPa), but it can tolerate pressures as high as 40 MPa. *P. abyssi *exists at the depth of about 2200 m (~22 MPa) but it can tolerate pressures up to 50 MPa [8]. These organisms differ markedly by the composition of utilized substrates. In contrast to *P. furiosus *and *P. abyssi, P. horikoshii *cannot grow on substrates with low content of peptides and amino acids. A probable reason why is the absence of appropriate operons, which renders *P. horikoshii *unable to synthesize many amino acids (Table 1). Lack of the chemotactic genes is characteristic of *P. furiosus *[5,9]. From a survey of the features of the habitats of these archaea (Table 1), it may be inferred that the main factors by which they differ are hydrostatic pressure (habitat depth) and specificity of utilized substrate. These archaea may be classified according to these factors as follows: *P. furiosus*, piezotolerant living in pressures near atmospheric; *P. horikoshii*, piezotolerant, living in moderate pressures, on amino acid-enriched substrates; *P. abyssi*, piezotolerant, living predominantly in deep seas. In the course of evolution, the three related species of Pyrococcus became adapted to life in various abiotic and biotic environments. With this in mind, a more thorough analysis of the molecular mechanisms at the genomic level providing this adaptation appears worthwhile [10].

**Table 1:** Ecology and genome organization of *P. furiosus, P. abyssi*, and *P. horikoshii *(based on [3-5,8,9,15,17,27,70,71])

	*P. furiosus *	*P. abyssi *	*P. horikoshii *
General ecological characteristics			

Doubling time (min) [15,70,71]	37	33	32

Pressure optimum (MPa) [8]	11	21	1

Pressure range (MPa) [8]	<0.1-25	<0.1-40	<0.1-35

Temperature optimum (°C) [15,70,71]	100	96	98

Temperature range (°C) [15,70,71]	70-103	67-102	<80--102

Salt concentration optimum (%) [15,70,71]	2	3	2,4

Salt concentration range (%)[15,70,71]	0.5-5	0.7-5	1-5

pH optimum [15,70,71]	7	6.8	7

pH range [15,70,71]	5-9	4-8.5	5-8

Carbohydrate and energy sources			

Complex substrates (i.e., yeast extract, peptone, etc.), 20 individual amino acids [15,70,71]	growth	growth	growth

Pyruvate, maltose [15,70,71]	growth	growth	death

Casamino Acids [15,17,70,71]	weak growth	growth	death

β-glucosides (i.e., cellobiose and laminarin) [27]	growth	death	death

Other requirements			

S^0 ^[15,70,71]	practically no growth	enhanced growth	enhanced growth

Tryptophan [15,70,71]	not needed	not needed	needed

Genome features			

Chromosome size (bp) [3-5]	1,908,256	1,765,118	1,738,505

G + C content (mol %) [3-5]	40.8	44.7	41.9

Number of ORFs [5]	2,208	1,765	2,061

Clusters of long tandem repeats [5]	7	4	6

Insertion sequences [5]	24	1	1

Amino acid biosynthesis (Val, Leu, Ile, Trp), aromatic amino acids biosynthesis, maltose transport, phosphate uptake [5,9]	yes	yes	no

Restriction/modification enzymes (protection from bacteriophages) [5,9]	no	yes	no

Chemotaxis-related genes [5,9]	no	yes	yes

Histidine biosynthesis, riboflavin biosynthesis, trehalose transport, citrate cycle, cobalt transport [5,9]	yes	no	no

In the current study, we analyze genomic variability in three Pyrococcus species with regard to changes in their environmental conditions during evolution. Emphasis is on factors of genome evolution, such as gene loss, radical to conservative amino acid replacement rate ratio. Analysis of radical (large changes in the physico-chemical properties) and conservative (small changes) amino acid fixations yields useful information about modes of protein evolution. An excess of radical over conservative substitutions is a significant indicator of evolution of the proteins under positive natural selection [11,12]. Here, we analyzed the evolution of the protein-coding genes in the archaea of the Pyrococcus genus to detect the gene families evolving under positive selection during adaptation of the Pyrococcus species to different abiotic and biotic conditions. As a result, we demonstrated that the function of the genes evolving under positive selection strictly depends on abiotic and biotic conditions to which *P. furiosus, P. abyssi*, and *P. horikoshii *became adapted.

As known, protein structure alters under the changes in pressure [13]. In fact, pairwise comparison of the shallow-water *P. furiosus *with the deep-water *P. abyssi *demonstrated that amino acid substitution is asymmetrical: arginine, serine, glycine, valine, and aspartic acid were more frequently, while tyrosine and glutamine were less frequently used in *P. abyssi *[14]. The inference was that the more polar amino acids are more piezophilic than the heavy ones [14]. In this work, we also analyzed the proteome evolution of the three species of the Pyrococcus genus in terms of tendencies toward fixation of amino acids with particular physicochemical properties. We disclosed that the changes in protein structure of Pyrococcus might have been related with optimization of hydrophobic core volume in the vast majority of proteins, but not with adaptive evolution of proteins with specific function.

Thus, pressure might not have been the sole agent that brought about adaptation of the *P. furiosus, P. horikoshii*, and *P. abyssi *genomes. For example, at the stage of the divergence of *P. horikoshii *and *P. abyssi*, change in the trophic level might have been a no less important evolutionary factor. *P. horikoshii *might have acquired a consumer status at a high level, whereas *P. abyssi *at a low one.

## Results and Discussion

### Pyrococcus phylogeny

**Figure 1:**
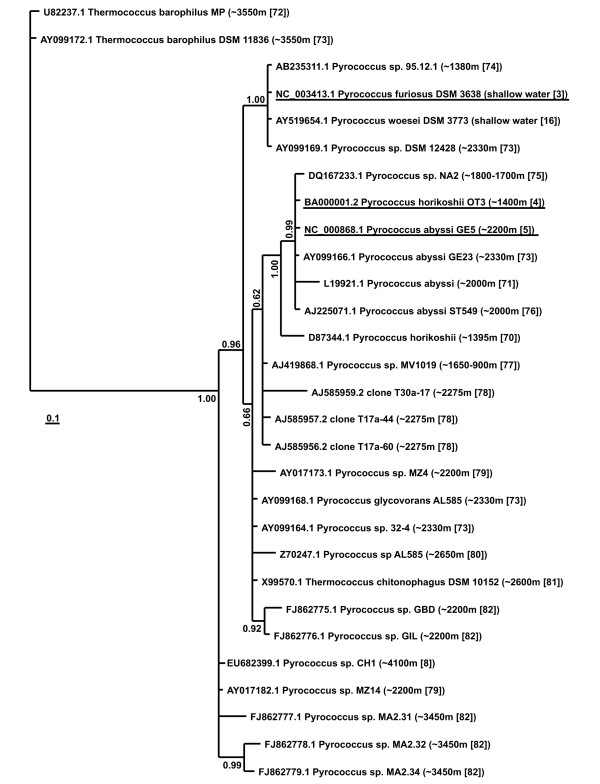
**Phylogenetic relationships among Pyrococcus strains (from **[3-5,8,16,70-82]) **based on the 16S rRNA gene sequences**. Bayesian posterior probabilities of nodes are shown.

A phylogenetic tree of archaea of the Pyrococcus genus derived from comparisons of 16S rRNA gene sequences is given in Figure 1. Organisms whose genomes have been completely sequenced are underlined. To get an idea of how these microorganisms are distributed according to depth, habitat depth is given to the right of each species name. Most archaea of this genus inhabit at the depth of 1300 - 2600 m. They also include inhabitants of shallow water (*P. furiosus *[15] and *P. woesi *[16]), and the obligate piezophile *Pyrococcus *CH1 [8]. *Pyrococcus *CH1 has been sampled at the depth of 4700 m; experimental data indicate that they die at pressures below 15 MPa, 50-60 MPa being optimal for their growth.

The data on phylogeny and habitat conditions allowed us to assume that the common ancestor of the *P. furiosus*, *P. horikoshii*, and *P. abyssi *might have inhabited at the sea depth of ~2000 m under high pressure (~20 MPa). Evolving from the common Pyrococcus ancestor, the branch with the *P. furiosus *ancestor separated. These organisms might have coped with life in shallow water and probably lost some of the functions providing survival under high pressure. The ancestor of *P. horikoshii *and *P. abyssi *might have remained tolerant to high pressure. However, *P. horikoshii *was less tolerant to high pressure and it became adapted to existence in an amino acid enriched environment (Table 1). *P. abyssi *remained in deep sea habitat where *P. horikoshii *and *P. abyssi *common ancestor occurred.

### Gene loss events in the Pyrococcus proteomes

We have analyzed a sample of 164 groups of orthologous genes *P. furiosus*, *P. horikoshii*, and *P. abyssi*, of which one of the three species underwent gene loss (Additional data file 1). To define the functional classes of genes most susceptible to changes and the organisms, which as a rule lose genes with these functions, we compared the deviation of the number of lost genes with the 1:1:1 uniform distribution in different functional groups for the three genomes. The χ^2 ^test was used to determine the significance of the deviation. The results are set out in Table 2. The data for gene loss in 164 groups of orthologous genes are in agreement with those reported elsewhere for comparisons of the complete Pyrococcus genomes (compare with Table 1). Table 2 clearly shows that gene loss according to function and organism are nonuniform. *P. furiosus *lost the chemotaxis and cell motility genes in the course of evolution; according to our classification, these are the genes referred to the functional group "signal transduction mechanisms" and "cell motility", respectively. Gene loss was dramatic in *P. horikoshii*. It concerned the genes for metabolism, those for energy production and conversion, transport and metabolism of carbohydrates and lipids. Taken together with the experimental data (Table 1), this allowed us to assign archaea of the *P. horikoshii *species to the group inhabiting enriched microbiological communities of hydrothermal vents, habitats saturated with amino acids, i.e. with a status of a high level consumer. In contrast, the number of gene losses during evolution is minimal in *P. abyssi*. Thanks to it, *P. abyssi *is able to exist in deficient aggressive environments [17] it is, probably, a consumer of a low level in microbiological communities of hydrothermal vents.

**Table 2:** Events of gene losses in the *P. furiosus*, *P. horikoshii *and *P. abyssi genomes*

Functional group	Number of genes lost			
		
	P. horiko-shii	P. abyssi	P. furiosus	p* (χ^2^)
INFORMATION STORAGE AND PROCESSING	9	2	10	0.066

Translation; ribosomal structure and biogenesis	1	1	3	0.449

Transcription	5	1	4	0.273

Replication; recombination and repair	3	0	3	0.223

CELLULAR PROCESSES AND SIGNALING	4	4	25	**0.000002**

Cell cycle control; cell division; chromosome partitioning	1	0	0	0.368

Defense mechanisms	1	1	5	0.102

Signal transduction mechanisms	1	1	11	**0.0004**

Cell wall/membrane/envelope biogenesis	1	0	4	0.074

Cell motility	0	0	3	**0.05**


Intracellular trafficking; secretion; vesicular transport	0	1	1	0.607

Posttranslational modification; protein turnover; chaperones	0	1	1	0.607

METABOLISM	34	9	21	**0.0007**

Energy production and conversion	11	1	5	**0.011**

Carbohydrate transport and metabolism	8	0	4	**0.018**

Amino acid transport and metabolism	3	1	0	0.174

Nucleotide transport and metabolism	5	2	4	0.529

Coenzyme transport and metabolism	3	4	2	0.717

Lipid transport and metabolism	3	0	0	**0.05**

Inorganic ion transport and metabolism	1	1	5	0.102

Secondary metabolites biosynthesis; transport and catabolism	0	0	1	0.368

FUNCTION UNKNOWN	23	13	10	**0.049**

It has been shown that the archaeal genes of the Pyrococcus genus are prone to horizontal transfer [7,18,19]. Six regions extremely variable because of multiple gene transfers from related species/strains have been distinguished in *P. furiosus *[19]. Horizontal gene transfer may affect statistical estimates of gene loss significance. This prompted us to perform additional studies to estimate the frequency of these events in the analyzed 164 gene clusters and to judge how this may possibly affect the statistical relation between gene functional class and gene loss event (Additional data file 1). To identify the cluster with the horizontally transferred genes, we used the HGT-DB database [20,21]. This database contains the genes from the genomes of various prokaryotes, which they might have been acquired through horizontal transfer. Of the 164 clusters with ongoing gene loss, 9 (~5%) only according to the HGT-DB data potentially contained the horizontally transferred Pyrococcus genes. Of these 9, 6 were members of the group with unknown functions, the other 3 we annotated as performing functions of "nucleotide transport and metabolism", "posttranslational modification, protein turnover, chaperones", and "cell motility". Of the three functional groups according to the data in Table 2, the "cell motility" group only shows statistically significant deviation from 1:1:1, with all the lost genes in the group belonging to *P. furiosus*. The gene with the "cell motility" function, in which horizontal transfer might have occurred (GenBank GI 14590358), belongs to the *P. horikoshii *species. It is of interest that the horizontally transferred genes in the "nucleotide transport and metabolism" (GenBank GI 14590010) "posttranslational modification, protein turnover, chaperones" (GenBank GI 14590433) groups belong to *P. horikoshii*, too. Thus, the events of horizontal gene transfer for the genomes we analyzed could not have significantly affected the identification of functional classes dramatically undergoing gene loss.

### Positive selection of the Pyrococcus protein-coding genes

**Figure 2:**
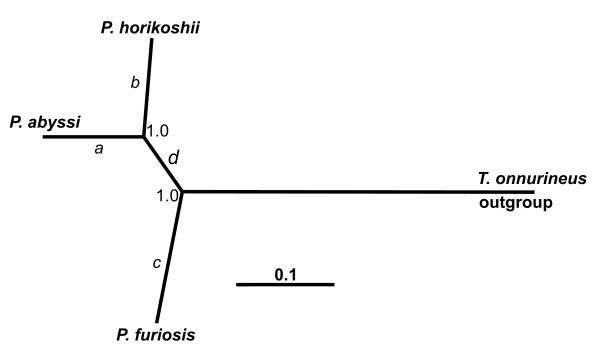
**Phylogenetic relationships among the P. abyssi, P. horikoshii, P. furiosus, and T. onnurineus genomes based on the concatenated amino acid sequences**. Bayesian posterior probabilities of nodes are shown. Ingroup branches are labeled *a *through *d*.

Search of genes evolving under Darwinian positive selection was performed for 911 orthologous clusters (Additional data file 2). For each cluster, there were 4 homologous sequences of genes encoding proteins of the three Pyrococcus species (*P. furiosus*, *P. horikoshii*, and *P. abyssi*) and one *T. onnurineus *species as outgroup in each cluster (see Methods). The phylogenetic tree for these four microorganisms built from concatenated protein alignments from the 911 clusters is shown in Figure 2. Our further analysis proceeded from this tree. We were mostly interested in the evolutionary events associated with change in environmental conditions for *P. abyssi, P. horikoshii*, and *P. furiosus *species as they diverged from common ancestors on the internal nodes of the tree. The tree branches representing the divergence are designated as *a*, *b*, *c*, respectively (Figure 2). The internal tree branch representing evolution from the common ancestor of *P. furiosus *and *T. onnurineus *to the recent common ancestor of *P. abyssi *and *P. horikoshii *is designated as *d*.

To analyze the evolution mode of the genes, we used the sequences reconstructed for the inner tree nodes (Figure 2). We detected positive selection modes by calculation of the radical over conservative replacement ratio for genes and of the pairwise γ-distances for proteins (see Methods section).

As a result, we identified a number of genes that underwent positive selection at various stages of the evolution of the Pyrococcus genus. Of these, the genes in 154 clusters underwent positive selection for the branch *a *(Figure 2); in 208 clusters for the branch *b*; in 131 clusters for the branch *c*; in 168 clusters for the branch *d *(Additional data file 3). Positive selection was not identified on one of the branches leading to the Pyrococcus species in 422 clusters. A part of the genes underwent positive selection on one of the branches might have also underwent positive selection on its other branches. However, we also detected genes, which underwent selection pressure on a single branch of the tree. We designated such clusters as "unique". The relative proportion of the total number of "unique" clusters was 48.7% (75 clusters) for the branch *a*, 49.5% (103 clusters) for the branch *b*, 51.9% (68 clusters) for the branch *c*, and 57.1% (96 clusters) for the branch *d *(Additional data file 3).

Further analysis was aimed at detection of functional classes whose genes mainly underwent positive selection on the indicated branches (Additional data file 3). We determined significance of associations between the evolution under positive selection and the functional class of genes using the permutation statistical test (see Methods). The test was applied to all the genes undergoing pressure of positive selection and to the genes undergoing it on a certain branch (the "unique" clusters). The results for each of the examined branches are presented in Table 3.

**Table 3:** Functional groups enrichment with clusters under different evolution modes

Functional group	Total clusters in group	All positively selected clusters on the Pyrococcus tree branches (as in Figure 2) †				Unique positively selected clusters on the Pyrococcus tree branches (as in Figure 2) †				Clusters not under positive selection †
			
		*a*	*b*	*c*	*d*	*a*	*b*	*c*	*d*	
Translation; ribosomal structure and biogenesis	168	32	45	48 ****	37	13	16	26 ****	19	55****

Transcription	62	13	25 ***	13	15	3	10	5	5	20**

Replication; recombination and repair	51	10	10	4*	6	6	6	3	5	26

Cell cycle control; cell division; chromosome partitioning	13	4	1*	1	1	2	0	0	1	8

Defense mechanisms	12	2	3	2	3	0	1	0	1	7

Signal transduction mechanisms	10	0	3	1	1	0	3	1	1	5

Cell wall/membrane/envelope biogenesis	19	2	1**	1*	2	2	1	1	2	13 *

Cell motility	11	3	2	3	2	1	2	1	1	4

Intracellular trafficking; secretion; vesicular transport	11	2	3	1	5 *	1	1	0	3	3*

Posttranslational modification; protein turnover; chaperones	32	6	8	2*	6	2	4	1	4	16

Energy production and conversion	78	13	14	10	12	5	9	8	5	40

Carbohydrate transport and metabolism	49	4*	10	6	8	2	6	2	6	28

Amino acid transport and metabolism	61	13	18	2***	10	7	9	2*	4	29

Nucleotide transport and metabolism	38	4	10	8	13 *	2	6	3	10 **	10**

Coenzyme transport and metabolism	58	12	17	8	11	5	9	4	5	23

Lipid transport and metabolism	15	1	2	0	3	0	2	0	2	10

Inorganic ion transport and metabolism	46	9	4**	4	4*	8 *	3	4	4	26

Secondary metabolites biosynthesis; transport and catabolism	7	2	1	0	2	2	0	0	1	3

Function unknown	170	22*	31*	17*	27	14	15	7*	17	96 **

Total	911	154	208	131	168	75	103	68	96	422

**† **- Bold denotes cases with excess of clusters (*0.01 <*p *≤ 0.05; **0.001 <*p *≤ 0.01;; ***1 × 10^-4 ^<*p *≤ 0.001; ****p ≤ 1 × 10^-4^), italics denote cases with lack of clusters (*0.01 <*p *≤ 0.05; **0.001 <*p *≤ 0.01; ***1 × 10^-4 ^<*p *≤ 0.001; ****p ≤ 1 × 10^-4^).

On the branch *a *leading to *P. abyssi*, there was a significant excess of genes whose positive selection was identified on that branch only (the "unique" genes) in the functional group "inorganic ion transport and metabolism" (Table 3), the great majority of these genes belong to the "multisubunit Na^+^/H^+ ^antiporter" family (Additional data file 4). It is of relevance that the genes for transmembrane inorganic ion transport in the abyssal microorganisms *Photobacterium profundum *and *Shewanella benthica *are mainly under positive selection [22]. Positive selection was not detected in the group of genes for this branch within the gene group accomplishing the functions of "signal transduction mechanisms", as for the functions of "carbohydrate transport and metabolism", the number of genes undergoing positive selection pressure proved to be smaller than expected to occur by random (Table 3). The branch *a *leads to *P. abyssi*, best adapted to life in high pressure environment (Table 1). Therefore, the relationship between positive selection and function of ion transport might have been caused by the microorganism response to high pressure environment. It should be noted that *P. abyssi *is closest to the possible common ancestor of the three Pyrococcus species in terms of habitat depth (see Table 1 and Figure 1). This could mean that this species did not experience sharp fluctuations in pressure and that positive selection might have been caused by alteration in substrate specificity [7,10]. In contrast to *P. horikoshii*, archaea of the *P. abyssi *species can grow on nutrient-depleted substrate (Table 1). Changes in the system of inorganic ion transport possibly took place during adaptation to nutrient-depleted environment. The inference that transport functions are of considerable importance to *P. abyssi *is also made from the small number of genes lost by this functional group (Tables 1 and 2).

Our analysis of the branch *b *leading to *P. horikoshii *showed that the genes for transcription machinery were mainly subject to positive selection events (Table 3). We annotated most of these *P. horikoshii *genes as encoding "transcriptional regulators" and various "subunits of DNA-directed RNA polymerase" (Additional data file 4). Changes in the transcription machinery might have been associated with the specific features of protein synthesis in this species. According to the data in Tables 1 and 2, *P. horikoshii *is characterized by substantial loss of the genes for metabolism, in particular loss of those for synthesis of aromatic amino acids. Probably, *P. horikoshii *was adapted to acquire a large number of amino acids from the external environment, and, therefore, lost need for autonomous synthesis of a number of amino acids. This simplification might have been resulted in changes within transcription machinery and, therefore, in reduction in energy costs of the biosynthesis of unnecessary proteins under certain circumstances [10]. We also identified functional groups in which positive selection pressure proved to be smaller than randomly expected. It is of interest that these are the functional groups "inorganic ion transport and metabolism" and "cell cycle control, cell division, chromosome partitioning" (Table 3). This fact shows that the evolution of these groups of genes took place in a neutral mode or under negative selection. Adaptation strategy to environmental conditions through chemotactic movement toward a source of nutrients (Table 1) might explain such a mode of evolution for the *P. horikoshii *genes for "inorganic ion transport and metabolism" and "cell cycle control, cell division, chromosome partitioning".

The branch *c *represents the divergence of the *P. furiosus *species, an inhabitant of shallow water, from its deep-sea ancestor. Prevalence of positive selection for the genes for "translation machinery" function is characteristic of this branch (Table 3). Various tRNA synthetases, for example Isoleucyl-, Alanyl-, Valyl-, Phenylalanyl-, Aspartyl/asparaginyl-tRNA synthetases, ribosomal proteins, also factors of translation initiation constitute the majority of the translation machinery genes under positive selection (Additional data file 4). It is of interest that changes in habitats for this particular branch result from a decrease in hydrostatic pressure caused mostly by a transition to novel shallow habitat (Table 1). There are lines of evidence indicating that change in hydrostatic pressure exerts an influence on ribosomal function both in *Escherichia coli *[23] and other prokaryotic organisms [24]. In fact, an increase in pressure leads to ribosome destabilization and dissociation [24,25]. This is the main reason why an increase in pressure is hazardous, even deadly to the cells [25]. It is also a fact that the ribosomal protein genes are among those highly expressed under these conditions [26]. It appears reasonable to assume that accelerated accumulation of radical substitutions in the genes for ribosomal functions may be associated with rearrangement of the ribosomal complex to stabilize it under normal pressure arisen after divergence from the deep-sea ancestors. It should be also noted that changes in the translation system may be due to specificity of biotic environmental conditions [10,27] to which *P. furiosus *adapts. This appears plausible because these organisms lack chemotactic adaptive mechanisms for nutrient concentrations in the environment, yet have genes for utilization of alternative sources of carbohydrate and energy for sustaining growth (Table 1). On the branch *c*, there was also a statistically significant deficiency in the number of positively selected genes of the functional group "replication, recombination and repair". This may be evidence of the operation of negative selection in this gene group, conservation of their functions designed to provide genetic material exchange of vital importance to the *P. furiosus *species [18,19].

The *d *branch corresponds to two internal nodes (Figure 2). It depicts the evolutionary pathway from the ancestors common to all the three Pyrococcus species to the one of the *P. abyssi *and *P. horikoshii *species. Analysis of positive selection events of the genes on this branch revealed that the positive selection is rather characteristic of the gene groups of "nucleotide transport and metabolism", "intracellular trafficking, secretion, and vesicular transport" (Table 3). Exemplary are the following ancestral genes for which positive selection was identified on this branch. They belong to the functional subgroups "nucleotide metabolism" (thymidine phosphorylase, adenylate kinase, cytidylate kinase, adenylate cyclase, purine nucleoside phosphorylase; see Additional data file 4) and "secretion" (these are either preprotein translocase or multiple antibiotic transporters; see Additional data file 4). Prevalence of the genes accumulating radical substitutions on this branch under the effect of positive selection in the system of nucleotide metabolism may indicate that metabolic DNA process might have been altered in the ancestor of *P. abyssi *and *P. horikoshii *species. This agrees with the experimental data indicating shorter doubling time of *P. abyssi *and *P. horikoshii *and also their smaller chromosome size compared with *P. furiosus *(Table 1). Overrepresentation of the number of the observed positively selected genes for "nucleotide metabolism" and those for "intracellular trafficking" appear to be consequences of the adaptation of the *P. abyssi *and *P. horikoshii *ancestor to the nutrition-depleted media. This inference is supported by the *P. abyssi *operon organization and requirements for nutrition (Table 1). Another observation was that the branch *d *is characterized by low frequency of genes subject to positive selection and performing the functions "inorganic ion transport and metabolism" (Table 3). It is of interest that a decrease in the frequency of these genes is characteristic also of the branch *b *representing divergence of *P. horikoshii *from the ancestor shared with *P. abyssi*. However, the evolutionary scenario is different for *P. abyssi*: this group of genes rapidly accumulated radical substitutions, as noted above. All this may be taken as evidence for negative (the branches *d*, *b*) to positive (the branch *a*) selection mode replacement at the stage of evolution from the ancestor shared with the three Pyrococcus species for this group of genes. This may result from sharp change in ionic composition for the *P. abyssi *environment or in the site these bacteria occupy in the trophic chain (transition to the low consumer level) [10].

The gene sets subjected to positive selection are different on every one of the branches. This is apparent from the conducted analysis (Table 3). However, these variations in sets do not fit closely the habitat depth-dependent variations in the three Pyrococcus species with respect to both the number of genes under selection and their functions. The phylogenetic data (Figure 1) indicate that changes in depth were greater in *P. furiosus*, *P. horikoshii *inhabits intermediate depths; as for *P. abyssi*, it is closest to the common ancestor in terms of habitat depth. Despite this the *P. abyssi *genome possesses some genes susceptible to positive selection. The number of the genes is comparable with that experiencing positive selection at the stage of *P. furiosus *divergence. An interesting fact is brought into prominence: the greater number of genes under positive selection in *P. horikoshii *than in the other two species (including the "unique"; Table 3, last line). This inconsistency becomes explicable under the assumption that, besides pressure, there might have been other factors altering in the Pyrococcus species and promoting faster substitution fixation in a number of genes. Changes in the types of consumed substrates appeared to be of no less importance than pressure in promoting substitution fixation (Table 1). The influence of other intervening factors, unknown as yet, cannot be ruled out. Having presumably lost its ability to synthesize a number of important amino acids, *P. horikoshii *was compelled to procure them externally. Interestingly, in *P. horikoshii *the number of genes under positive selection is larger in those groups that are involved in production and transport of metabolites ("transport and metabolism", Table 3, lines 11-16). A significant prevalence of positive selection for the "inorganic ion transport and metabolism" function was observed for *P. abyssi*. In evolving *P. abyssi *and *P. horikoshii*, substrate preference presumably shifted stepwise both on the branch *d *(significant prevalence of positive selection for the "nucleotide transport and metabolism") and after divergence (on the branches *a *and *b*, as Figure 2 shows). *P. furiosus *presumably retained the substrate preferences of the ancestor the three Pyrococcus species shared. To summarize, the three Pyrococcus species might have diverged under the impact of two consequential factors: changes in the habitat depth and substrate specificity. *P. abyssi *persisted in occupied depths, although forced to partly alter certain substrate specificities. In *P. furiosus*, substrate specificity of the common ancestor remained unaltered, habitat depths changed. In *P. horikoshii*, depth of habitat and specificity of consumed substrate both underwent changes. This may be a plausible reason why gene number under positive selection is larger in *P. horikoshii *compared to the other two Pyrococcus species.

Statistical analysis of the association between the negatively and neutrally evolving genes and their function demonstrated that positive selection is less targeted at functional groups, such as "cell wall/membrane/envelope biogenesis" (Table 3). It should be emphasized that comparison of microorganisms of the *Shewanella benthica *yielded the reverse: many of the genes for "membrane function and structure" were found to be subject to positive selection [22]. Indeed, the cell membrane is one of the structural elements of cells presumably the most severely affected by high pressure [28]. Nevertheless, Pyrococcus species are thermophilic archaea with membrane structure different from bacterial. The chemistry of their lipid membranes is based on poly-isoprene. The isoprene side chains have methyl groups. The protruding methyl groups maintain the biologically active state of the membranes even at high pressure [29-31]. Moreover, the ends of the two poly-isoprene chains can be linked chemically, forming lipid monolayer. Such a membrane structure characteristic of archaea is already optimal for cell existence in extremely high temperatures and pressures. As a result, its response to change in hydrostatic pressures may not demand essential molecular rearrangements and this precluded accelerated accumulation of radical substitutions in the evolving species of the Pyrococcus genus.

We also investigated how events of horizontal gene transfer may affect the estimates for the association between genes function and positive selection mode of their evolution. To this end, we calculated the frequencies of horizontal gene transfer on the branches *a*, *b*, and *c *(Figure 2) for clusters of homologous genes for which positive selection was identified. The number of clusters with possible horizontal gene transfer was determined as: 16 (10.4%) clusters for the branch *a*; 14 (6.7%) clusters for the branch *b*; 12 (9.2%) clusters for the branch *c*; and 20 (11.9%) clusters for the branch *d*. Of 35 clusters (8.3%) with horizontal gene transfer events, positive selection was not identified on any branches. The data for horizontal transfer events (see Additional data file 5) show that the number of clusters with identified horizontal transfer events is comparatively small (97, ~10%). However, from the data tabulated in Additional data file 5 it follows that the gene clusters with the horizontal transfer event are nonuniformly distributed among the functional groups. Larger frequency of horizontal gene transfer was identified for the functional group "replication, recombination and repair" on the branch *a *leading to *P. abyssi *and on the branch *d *leading to the ancestor of *P. abyssi *and *P. horikoshii *(Additional data file 5). The functions of genes of the group "replication, recombination and repair" subject to horizontal transfer are predominantly related to the life cycle of various bacteriophages (Additional data file 2). Bacteriophages are important gene-transfer vehicles, owing to their great abundance and the ability to insert themselves into chromosomes as prophages without causing cell lysis, thereby altering the gene content of their hosts [32]. Thus, the detected association between horizontal gene transfer and gene function appears reasonable. We also established statistical significance of the association between clusters of positively selected genes on the branch *b *involved in cell motility (Additional data file 5). One explanation may be that *P. horikoshii *demands enrichment of substrates with amino acids and that the systems of cell motility may be needed for the organism to relocate to this enriched environment.

The mechanism of *P. furiosus *adaptation to environmental conditions appears to be closely related with extensive transfer of genetic material. This is consistent with the recent data indicating that shuffling of *P. furiosus *genetic material is caused by mobile genetic elements [18,19,32,33]. It is of interest that, when clusters characterized by horizontal gene transfer were omitted from the group "translation, ribosomal structure and biogenesis" on the branch *c*, the statistical significance of excess of positively selected genes in this group remained in the range of *p *< 0.001.

### Detection of changes in the physicochemical properties of proteins on branches of Pyrococcus phylogeny

**Figure 3:**
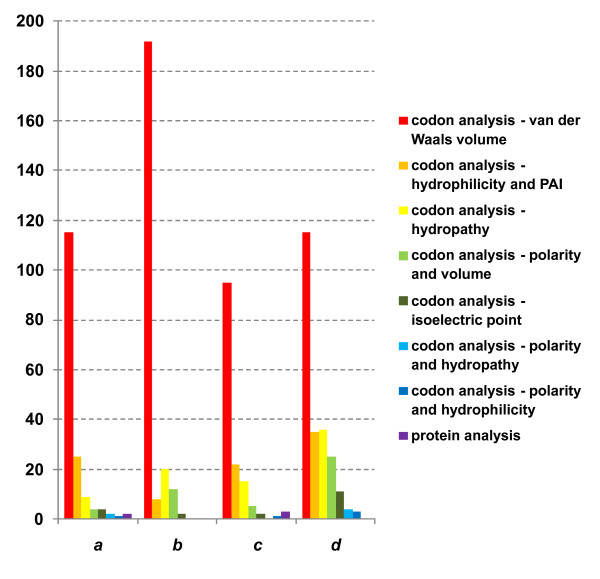
**Number of gene clusters for which positive selection was detected by different approaches and different amino acid physicochemical categorizations**. The bar colors for different approaches are shown on the right; *y*-axis: number of clusters; *x*-axis: branches (designations are as in Figure 2).

Analysis of the radical, *d*_*R*_, over conservative, *d*_*C*_, replacement rate ratio, which relies on amino acid classifications, gives important information about how the physicochemical properties of amino acids might have changed during evolution. To classify amino acids, we used a number of physicochemical characteristics and their pairs (7 classifications in all, see Methods). As a result, we obtained statistics for significant excess of radical substitutions over conservative with respect to ways of amino acids grouping. Figure 3 represents histograms for the number of homologous gene clusters in which *d*_*R *_was in significant excess over *d*_*C*_. From this figure, it is apparent that in most cases this excess is due to grouping of amino acids according to the side-chain van der Waals volume. A smaller number of genes with significant excess of *d*_*R *_over *d*_*C *_were identified when grouping was based on amino acid properties such as hydropathy and asymmetry during amino acid substitution, depending on hydrostatic pressure (Figure 3). The positive selection that was mainly related to change in amino acid side-chain volume may be due to optimization of the protein structure to maintain archaeal vital activities under various pressures.

The volume of the side-chain is a property that reflects structural characteristics of amino acids. Of the factors we considered here, making Pyrococcus species different from each other (Table 1), change in pressure may affect strongest protein structure. As known, increase in pressure can lead to destabilization of protein globule by water penetration into the inner hydrophobic core of protein through pores [28]. Substitutions causing volumetric changes may be targeted at tighter packing of the inner hydrophobic core, making it less susceptible to access of water molecules. The question is: What type of radical substitutions is needed for packing of the hydrophobic core of a protein to be tight? It would seem that the radical amino acid substitutions may have a destabilizing effect on the core protein structure (for example, replacement of amino acid with small side-chain by amino acid by a large one). However, small displacements of the elements of the protein secondary structure do make the hydrophobic core sufficiently flexible and provide efficient compensations for side-chain size variations [34-37]. As a consequence, even radical amino acid replacements (according to the side-chain volume classification) because of compensation do not produce "cavities" in the core, while the core becomes more tightly packed.

We have also followed trends for changes of the physicochemical properties on the branches of the phylogenetic tree on the basis of estimated occurrence frequencies of amino acids in Pyrococcus proteins and reconstructed sequences in the internal tree nodes. For this purpose, we used sample I (see Methods) composed of 855 protein clusters in which protein ancestral sequences were accurately reconstructed (Additional data file 6).

**Figure 4:**
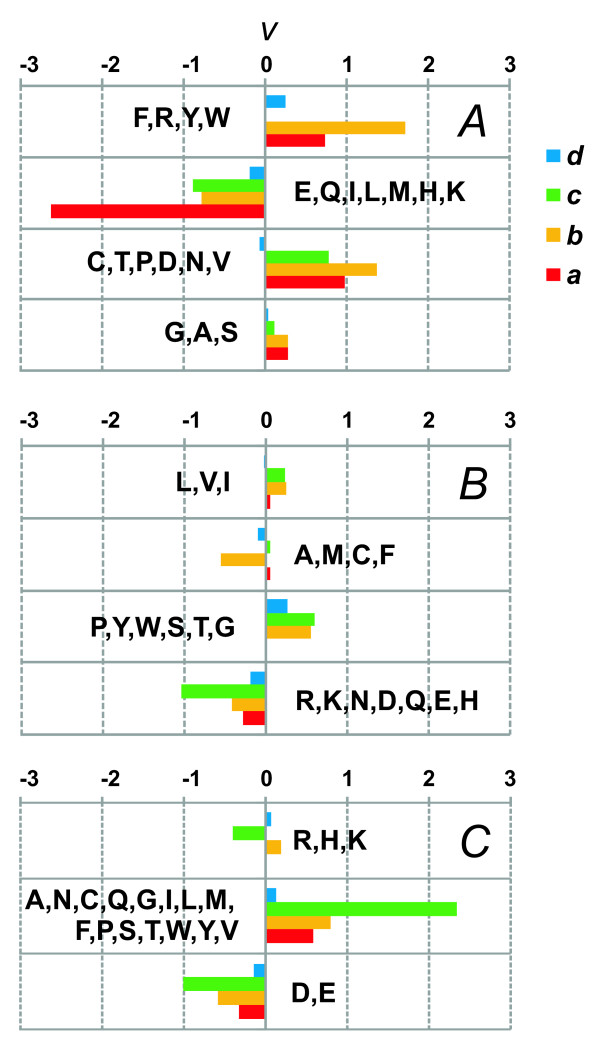
**The values of the ν parameter reflecting changes in amino acid frequencies for different evolutionary tree branches for categorizations based on the van der Waals volume, hydropathy and isoelectric point**. *y*-axis, amino acid groups (see Table 4); *x*-axis, *v *(see Methods section); branches are labeled as in Figure 2. Property designations: A, van der Waals volume; B, hydropathy; C, isoelectric point.

Figure 4 shows the relative change in the occurrence frequency of amino acids grouped according to their hydropathy, isoelectric point, and side-chain van der Waals volume. It is remarkable that during evolution of the proteins of the three Pyrococcus species there prevailed loss of amino acids belonging to the class of small van der Waals side-chain volume (E, Q, I, L, M, H, K; Figure 4A). The most prominent changes in the class of amino acids with small side-chain were characteristic of the branch *a *leading to *P. abyssi*. For the amino acids classes with tiny, medium and large side-chain volume, in contrast, the proportion of clusters in which their frequencies increase is greater (Figure 4A). This is in good agreement with the above results: radical replacements classified by amino acid side-chain volume contribute most to positive gene selection. They may provide efficient packing of amino acid side-chains in protein structure.

The very small change in the occurrence frequency of amino acids with tiny, small, medium, and large side-chain volume on the branch *d *leading from the ancestor of three Pyrococcus species to the ancestor of *P. abyssi *and *P. horikoshii *stirs interest (Figure 4A). This is possible evidence that the ancestor of *P. abyssi *and *P. horikoshii *might have evolved in unaltered environmental conditions.

The results shown in Figures 4B and 4C demonstrate that on the branch *c *leading to the archaea *P. furiosus*, a decrease in the occurrence frequencies of amino acids of the hydrophilic group (R, K, N, D, Q, E, H; Figure 4B) and an increase in those neutral (A, N, C, Q, G, I, L, M, F, P, S, T, W, Y, V; Figure 4C) are characteristic of the majority proteins. Such replacements might have played a role in the increase in protein hydrophobic core stability in *P. furiosus *[38-42] evolving on the branch *c*, because change in hydrostatic pressure is the most consequential factor affecting protein stability on this branch (Table 1).

According to the data in Figure 4C, the frequencies of amino acid class with medium value of the isoelectric point continuously increase on all the Pyrococcus branches, with the increase rate being proportional to the width of the spectrum of hydrostatic pressures at which *P. abyssi*, *P. horikoshii *and *P. furiosus *inhabit (Table 1). This harmonious trend for amino acid substitutions is explicable by an evolutionary pattern common to all branches. This is another argument in favor of the gradual adaptation of the species of the Pyrococcus genus to conditions of variable and relaxed hydrostatic pressures (Table 1).

**Figure 5:**
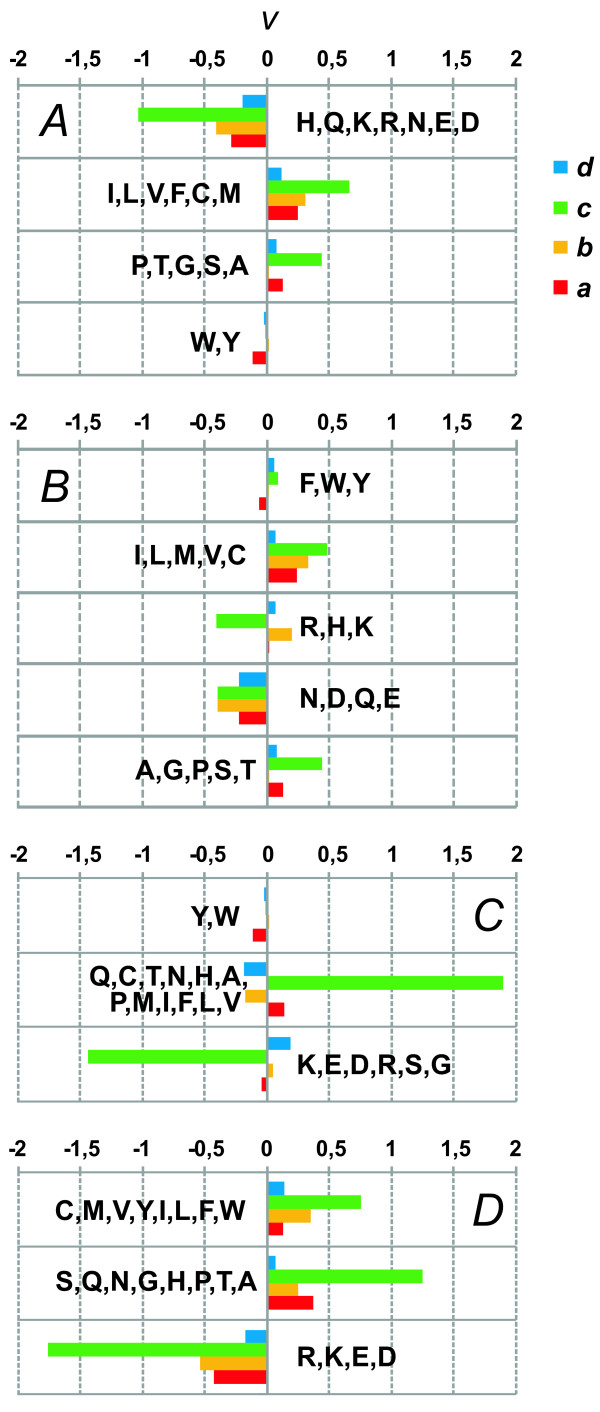
**The values of the ν parameter reflecting changes in amino acid frequencies for different evolutionary tree branches for categorizations based on the physico-chemical properties combinations**. Designations are as in Figure 4. Combination: A, polarity and hydropathy; B, polarity and volume; C, hydrophilicity and pressure asymmetry index; D, polarity and hydrophilicity.

Figure 5 shows the trends for changes in the occurrence frequencies of amino acids grouped by pairs of physicochemical properties. It is of interest that widest variability among all properties and all tree branches is characteristic of amino acids grouped according to hydrophilicity and the hydrostatic pressure asymmetry index, PAI (Figure 5C, D). The PAI value for amino acids have been estimated from comparisons of amino acid sequences from *P. furiosus *and *P. abyssi*; they reflect trends for amino acid substitution in shallow (*P. furiosus*) and deep-water (*P. abyssi*) organisms [14,43]. Amino acids with the high PAI value occur more frequently among deep-sea species. Figure 5C shows that the widest variations in these characteristics reside on the branch *c*, which corresponds to *P. furiosus *divergence along the changes in the trends for the two classes of amino acids, one lost (Q, C, T, N, H, A, P, M, I, F, L, V), the other acquired (K, E, D, R, S, G) during adaptation to high pressure environment.

Figures 4 and 5 highlight an interesting feature. The greatest *v *values on these graphs are characteristic of the branch *c*. As noted, greatest change in the parameter of the Pyrococcus species environment such as hydrostatic pressure is on this branch (Table 1). The *v *values on the *c *branch for some of the classifications proved to be 5 times greater than on the branches *a*, *b*, and *d*. This parameter is characterized by variability in proteins at the level of single amino acid replacements. Major causes were the structural features of proteins from the families we examined (because the relative size of the functional protein sites is small and the proportion of replacements affecting protein functional regions proves to be small). Therefore, the data for the branch *c *may be evidence that in the course of evolution in response to change in hydrostatic pressure, proteins accumulated amino acid substitutions maintaining stability of the protein globule. In contrast, the relative rates in changes of frequencies in different amino acid classes on the branch leading to the *P. abyssi *and *P. horikoshii *ancestor is minimal, as clearly seen in Figure 5A, B and 5D. This, in turn, may be due to the slight change in biotic and abiotic conditions in the evolving ancestor of *P. abyssi *and *P. horikoshii*. This fits well in the above proposed model for changes in the habitats of the three species of the Pyrococcus genus based on the phylogenetic rRNA gene sequence data (Figure 1).

## Conclusions

Here we report the results of a study of the genomes and proteomes of the marine archaea of the Pyrococcus genus performed to identify features of their adaptation to the environment at the genomic level. Phylogenetic analysis based on 16S rDNAs suggested that the common ancestor of *P. abyssi, P. horikoshii *and *P. furiosus *might have inhabited at the sea depth of ~2000 m under high pressure (~20 MPa). After divergence from the common ancestor, *P. furiosus *became adapted to the conditions of shallow water, whereas *P. horikoshii *and *P. abyssi *ancestor presumably remained in deep seas. *P. horikoshii *and *P. abyssi *diverged later. *P. horikoshii *evolved features enabling it to adapt to life at medium and small depths. In contrast, *P. abyssi *presumably retained its ability to survive in the deep-sea habitats.

Analysis of gene loss and Darwinian positive selection on the tree branches representing the divergence of the Pyrococcus species from their common ancestor allowed us to reveal the functional group of genes more susceptible to gene loss and positive selection. The important role of hydrostatic pressure to which archaea adapted during divergence of *P. furiosus *from the common ancestor of *P. abyssi, P. horikoshii *and *P. furiosus *species is left certain. However, explanation of our results could not be reduced to change in hydrostatic pressure during habitat transitions as the sole causative evolutionary factor. With reference to the divergence of the ancestor of *P. horikoshii *and *P. abyssi *from the common ancestor, and also the divergence of *P. horikoshii *and *P. abyssi *from each other, a no less important evolutionary factor may be their transition to new roles in bacterial community. For example, *P. horikoshii *acquired the ability to live on amino acid enriched substrates and occupied a higher level consumer role; *P. abyssi *could survive on amino acid depleted substrates and presumably occupied a lower level consumer role.

The increasing number of sequenced prokaryotic genomes has promoted studies of the molecular mechanisms of microorganism adaptation to the environment at the genomic level. Approaches relying on gene search undergoing positive selection [7,11,22] appear promising. However, great difficulties may arise in distinguishing the relatively small number of factors of environmental variability that contribute to adaptation [10]. These difficulties are due to the extreme genomic plasticity of prokaryotes to fluctuations in their environment [7,10]. While indisputable for the *P. furiosus *species we examined, hydrostatic pressure alone did not contribute to their adaptation. Breakthroughs in understanding of adaptation of marine archaea will hopefully come with further sequencing and comparative analysis of the genomes of closely related microorganisms whose habitats differ by a single or just a few environmental factors.

## Methods

### Phylogenetic analysis of piezophilic Pyrococcus species

Phylogenetic analysis was performed to elucidate the relationships between the species of the Pyrococcus genus inhabiting under different hydrostatic pressures. For this purpose, 16S rRNA gene sequences from 26 Pyrococcus strains and a *Thermococcus chitonophagus *strain deposited in the GenBank database (release 171.0) were used [44]. 16S rRNA gene sequences from two strains of *Thermococcus barophilus *served as the outgroup.

Multiple 16S rRNA genes alignment (Additional data file 7) in the orthologous cluster was performed manually. We then inferred phylogenetic trees by the Bayesian method using the MRBAYES 3.1.2 program [45]. Bayesian phylogenetic inference was made under Hasegawa-Kishino-Yano model (nst = 2) of nucleotide substitutions [46], rates was varied across nucleotide sites according to the gamma model, the model also takes into account the proportion of invariable sites (rates = invgamma).

### Analysis of molecular evolution of the Pyrococcus genomes

#### Genome sequences

Analysis of the evolutionary mode of the Pyrococcus genes and proteins was based on complete genome sequences from the *P. furiosus, P. horikoshii*, and *P. abyssi *(Table 1), also on those from *T. onnurineus *deposited in the GenBank database (release 171.0) [44]. We used as outgroups genes and proteins of the archaea *Thermococcus onnurineus*, a hyperthermophile that inhabits deep-sea water [47]. The *T. onnurineus *genome is closely related to the genomes of the Pyrococcus genus, it is of 1,847,607 bp and contains 1,976 CDSs of which about 1,100 are shared with the *P. furiosus *and *P. abyssi *species [47].

#### Identification of orthologous clusters, multiple protein and DNA alignment

We grouped the proteins according to their sequence similarity in the clusters using the BLASTCLUST program from the BLAST 2.2.19 package [48] with threshold similarity greater than 50%. We obtained 911 orthologous clusters containing only one homologous gene from each of the four species (Additional data file 2). It should be cautioned that the proposed approach does not make possible the detection of the complete set of orthologous genes in the genomes under study. Some orthologous relationships may be elusive, for instance, the similarity between fast evolving orthologous sequences may fall below the 50% threshold. Orthology in the strict sense can be defined by using a special algorithm, one of the kind Tatusov et al. [49] developed. The present analysis was to meet two less strict conditions: (1) the structure of homologous proteins should be highly similar (this was ensured by a 50% sequence similarity threshold); (2) at this similarity level, the protein in the related organism should not contain duplicated homologs. In fact, duplications can affect gene function and result in accelerated substitution rate in one of the homologs [50]. Our aim here was to detect genes under positive selection as a result of organismal adaptation to novel environment, not of acquirement of novel function.

Multiple alignments of protein sequences from the orthologous clusters were obtained using the MAFFT 6.704b program [51]. Codon sequence alignments were based on the protein sequence alignments.

Additionally, we chose 164 orthologous clusters containing only one homologous gene from each of the three species (from *T. onnurineus *species or any one of the *P. furiosus, P. horikoshii*, and *P. abyssi *species), and used them in further analysis of the functional gene groups most severely affected by gene losses in the evolving Pyrococcus species (Additional data file 1).

#### Proteome phylogeny reconstruction

Striving to more accurately define phylogenetic relationships in the *P. furiosus, P. horikoshii*, *P. abyssi *and *T. onnurineus *species group, we used the concatenated multiple alignment from the 911 orthologous clusters. We inferred the phylogenetic tree using the MRBAYES 3.1.2 program under a mixed model of amino acid substitutions (aamodelpr = mixed) [52], rates were varied across protein sites according to the gamma model that also takes into account the proportion of invariable sites (rates = invgamma). The CpREV empirical model of amino acid substitution for proteins [53] was the most appropriate for description of the evolution of the examined protein sequences (posterior probability of the CpREV model = 1.0).

#### Functional annotation of protein clusters

Function of every orthologous cluster of proteins was determined manually using the arCOG [54], KEGG Orthology (release 50.0) [55], InterPro (release 21.0) [56] θ PFam (release 23.0) [57] databases. The orthologous clusters were grouped according to function on the basis of functional classification in the arCOG [54] database.

### Detection of positive selection on tree branches

The idea was to detect the genes subject to Darwinian positive selection as Pyrococcus species adapted to altered environmental conditions. We kept in mind that different amino acids in proteins bear different structural and functional loads [58,59]. To exclude putative neutrally evolving protein sites, before starting the detection of positive selection, we discarded columns of codons containing gaps and columns coding for the different three amino acids from the three species of the Pyrococcus genus.

In search of the genes and proteins subject to positive selection in the evolving Pyrococcus species, corresponding to the different branches of their phylogenetic tree, ancestral sequences were reconstructed in the inner nodes of the tree. We were aware that the best accuracy in reconstruction of ancestral protein sequences is achieved at the identity level of not less than 70% [60]. Accordingly, 911 four-species orthologous clusters were assigned to: sample I, 855 clusters in which three sequences belonging to the Pyrococcus species showed >75% identity for the first and second codon positions; or sample II, 56 clusters in which the sequences of Pyrococcus species showed ≤ 75% identity for these codon positions. Then, sample I clusters were analyzed on the basis of codon alignments and sample II on that of protein alignments. The ANC-GENE [60] program was applied to reconstruct ancestral sequences of proteins and genes. This software first infers the ancestral amino acid sequences using the distance-based Bayesian method under the given empirical model of amino acid substitution (CpREV model) and then the ancestral codon sequences under the restriction of the inferred ancestral amino acids. It was previously shown that the distance based Bayesian method of the ancestral amino acid sequences inference is just as accurate in ancestor reconstruction as the method of maximum likelihood, differing favorably from it in fastness and more modest resource requirements [60].

Within sample I clusters, the mean fraction of alignment columns coding for three different amino acids of the three Pyrococcus species was very small, 3.56%. After discarding of such alignment columns, the ancestral genes and proteins were reconstructed. The significance of reconstruction of sample I ancestral sequences was high, the mean posterior reconstruction probability of the amino acid sequence of the Pyrococcus species ancestor (the most distant ancestor) was 0.978 ± 0.034 (mean ± 2 standard deviations). After reconstruction of the ancestral gene sequences, the mean occurrence frequencies of single nucleotide substitutions within a codon were 19.63% for the branch *a*, 35.11% for the branch *b*, 39.49% for the branch *c*, 10.88% for the branch *d *(Additional data file 8). The mean occurrence frequencies of double and triple nucleotide substitutions within a codon were 5-7 times smaller. The estimates were 3.53% for the branch *a*, 4.61% for the branch *b*, 6.78% for the branch *c*, 2.13% for the branch *d *(Additional data file 8). These features of the sequences allowed us to perform positive selection detection within sample I clusters by the radical, *d*_*R*_, over conservative, *d*_*C*_, nonsynonymous fixation rate ratio.

The  ratio requires carefully reconstructed ancestral protein sequences [61,62]. The assumption underlying this ratio is that conservative amino acid substitutions, which do not significantly affect the physicochemical properties of amino acid side-chains, are selectively neutral. In our case, the major advantage of the  measure is its insensitivity to synonymous substitution saturation [12]. There was synonymous substitution saturation effect for sample I clusters in the vast majority of pairwise gene comparisons. Moreover, synonymous changes are not silent in *E. coli *[63], also in a number of extremophilic prokaryotes [64]. The disadvantage of the  measure is its sensitivity mainly to unacceptably great GC- or AT-codon frequency bias (1:4) [65]. Nevertheless, the magnitude of the bias affecting the  appears to be insignificant, with a maximum bias of about 15% under the wide range of natural mutation parameters, and for positive selection to be inferred in practice one would require values of  > 1.3 [65]. It is of importance that our analysis did not identify unacceptably great bias of GC- or AT-codon frequencies (Additional data files 6 and 8). The  ratio for the two sequences was estimated by the HON-NEW program [62]; the transition-transversion rate ratio needed for HON-NEW was estimated by the YN00 program from the PAML package [66]. Substitution conservation was determined by comparing the biochemical properties of the side-chain of the corresponding amino acids. Calculation of the  was based on 7 classifications of amino acid physicochemical properties retrieved from the AAindex database [67], as shown in Table 4. Positive selection on the branches was considered as detected, if (1)  and , where *Var*(*d*_*R*_) and *Var*(*d*_*C*_) were the variances of *d*_*R *_and *d*_*C*_, respectively; (2) *d*_*C *_= 0 and *d*_*R *_>> 0 were found for any one of the amino acid classifications.

**Table 4:** Amino acid groups used in positive selection detection

Normalized van der Waals volume [83]	
Tiny [0; 1.6]	G, A, S
Small [2.43; 3]	C, T, P, D, N, V
Medium [3.78; 4.77]	E, Q, I, L, M, H, K
Large [5.89; 8.08]	F, R, Y, W
Hydropathy index [84]	
I [-4.5; -3.2]	R, K, N, D, Q, E, H
II [-1.6; -0.4]	P, Y, W, S, T, G
III [1.8; 2.8]	A, M, C, F
IV [3.8; 4.5]	L, V, I

Isoelectric point [85]	
I [2.77; 3.22]	D, E
II [5.05; 6.3]	A, N, C, Q, G, I, L, M, F, P, S, T, W, Y, V
III [7.59; 10.76]	R, H, K

Hydrostatic pressure asymmetry index, PAI [14] & Hydrophobic parameter [86]	
I	K, E, D, R, S, G
II	Q, C, T, N, H, A, P, M, I, F, L, V
III	Y, W

Polarity [87] & Hydrophobic parameter [86]	
I	R, K, E, D
II	S, Q, N, G, H, P, T, A
III	C, M, V, Y, I, L, F, W

Polarity & Volume [87]	
I	A, G, P, S, T
II	N, D, Q, E
III	R, H, K
IV	I, L, M, V, C
V	F, W, Y

Polarity [87] & Hydropathy index [84]	
I	W, Y
II	P, T, G, S, A
III	I, L, V, F, C, M
IV	H, Q, K, R, N, E, D

Within the sample II clusters, the mean fraction of alignment columns coding for the three different amino acids in the three Pyrococcus species was great, 12.4%. The accuracy of the reconstruction of the ancestral protein sequences after omission of such alignment columns was significantly poorer than in sample I (the mean posterior reconstruction probability of the amino acid sequence of the Pyrococcus species ancestor was 0.93 ± 0.04). After reconstruction of the ancestral gene sequences, the mean occurrence frequencies of single nucleotide substitutions within a codon became comparable for sample I on all the tree branches (Additional data file 8). As for the mean occurrence frequencies of double and triple nucleotide substitutions within a codon, it became twofold greater than for sample I, making up 8.21% for the branch *a*, 7.38% for the branch *b*, 13.74% for the branch *c*, and 5.24% for the branch *d *(Additional data file 8). For this reason, it was decided to identify the positive selection in sample II clusters only on the basis of calculation of the pairwise protein-protein γ-distances (Γ) between the ancestral and extant proteins [68]. Positive selection on the branch was considered as detected, if Γ_*i *_- *Var*(Γ_*i*_) ≥ 0.45 under the condition that Γ_*i *_- *Var*(Γ_*i*_) = Γ_max _- *Var*(Γ_max_), where *Var*(Γ_*i*_) was the variance of Γ_*i *_distance at the branch *i*, *Var*(Γ_max_) was the variance of the Γ_max _distance that corresponded to the longest tree branch of the set of tree branches *R *∈ [*a*, *b*, *c*, *d*].

### Detection of relation between evolution mode and protein function

To estimate the relation between evolution mode (for example, positive selection mode) and protein function, the permutation statistical test was used. Functional class was determined for each protein cluster, as described above. Based on analysis of the evolution mode, it was determined whether genes (proteins) of each cluster evolve in the positive selection mode (*PS *= 1) or not (*PS *= 0) on a certain tree branch. As a result, the significance of the occurrence of the positive selection mode for a cluster of a particular functional class was established (*PS *= 1). For this purpose, in a sample of 911 clusters, the number of clusters assigned to a particular functional class (*Class*) and evolving in the positive selection mode on a certain tree branch, *n*(*Class *+ *PS*), was calculated. Then, 10^5 ^samples were generated by random permutation of the *PS *values for this cluster set. For each random sample, the number of *n*_*Rand *_(*Class *+ *PS*_*Rand*_) clusters, which belonged to a given functional class and had *PS*_*Rand *_= 1, was estimated. During the test we calculated the number of random samples M, in which *n*_*Rand *_(*Class *+ *PS*_*Rand*_) > *n*(*Class *+ *PS*). The M/10^5 ^value expressed the probability *p*, at which the occurrence of the *Class*+*PS *observed in the initial cluster sample may arise randomly. The relation between functional class of clusters and evolution in the positive selection mode on a tree branch was considered to be significant, if *p *< 0.05. This test is an analog of Fisher's exact test [69]. Using these data, we can also estimate the probability *p*' = 1-M/10^5 ^at which the occurrence of the *Class*+*PS *did not occur in the initial cluster sample. If this value is significantly small (< 0.05), this may indicate that the genes from the corresponding functional class did not evolve under positive selection and mostly evolved neutrally or under stabilizing selection.

### Detection of amino acid change direction

The detection of evolutionary change in protein properties on each branch of the phylogenetic tree of the *P. furiosus, P. horikoshii*, and *P. abyssi *species was determined for each homologous protein cluster, *n*, by calculating the *f*_*i*,*n *_values expressing the occurrence frequency of a particular amino acid group, *i *(for example, *i *is the occurrence frequency of the amino acid group "G, A, S", Table 4).

These frequencies were determined in the nodes that corresponded to a particular phylogenetic tree branch (*a*, *b*, *c*, *d*, Figure 2). From the known occurrence frequency of the amino acid group *i *in the ancestral node (the amino acid groups are listed in Table 4), , and in the offspring node, , for the *n*-th cluster, the direction of change in the amino acid group *i *on the tree branch *j *was determined as . If the frequency for amino acid group *i *is greater in offspring sequence, the  value will be smaller than zero. And vice versa, if the frequency for the amino acid group *i *in the course of evolution from ancestor to offspring decreases, then  >0. To numerically estimate prevalence of a direction in amino acid change on the branch *j*, the following was calculated: the number of  clusters with an increase in the occurrence frequency of the amino acid group *i *( < 0), the number of  clusters with a decrease in the occurrence frequency of the amino acid group *i *( > 0), also , the number of clusters with an unaltered frequency of group *i *( = 0). The  relation was taken as the measure of prevalence of a direction of evolutionary reorganization in the amino acid group *i *on the *j *phylogenetic tree branch. If  = 0, then on the branch *j *the number of proteins in which the occurrence frequency of the amino acid group *i *increased was equal to the number of proteins in which those of the amino acid group *i *decreased; if  > 0, the occurrence frequency of the amino acid group *i *increased, and the reverse, if  < 0, a decrease in the occurrence frequency of the amino acid group *i *occurred.

### Possible horizontal transfer detection

We used the information stored in the Horizontal Gene Transfer Database, HGT-DB [20,21], for detection of possible horizontal transfer of genes in the species *P. furiosus*, *P. horikoshii*, and *P. abyssi*. The relation between possible horizontal gene transfer operating on the phylogenetic tree branches and gene/protein functional group was analyzed by the permutation statistical test, as described above.

## Authors' contributions

KVG performed the phylogenetic analysis, detection of positive selection, analysis of protein evolution, suggested the causes for positive selection events. DAA initiated, designed and coordinated the study, performed the initial analyses of genes and proteins, proposed the idea for the relation between gene positive selection and protein evolution. NAK initiated the bacterial genome study, and participated in its coordination. All authors read and approved the final manuscript.

## Supplementary Material

Additional file 1 **MS Excel file containing annotations of proteins of the 164 orthologous clusters from the *P. furiosus*, *P. horikoshii*, *P. abyssi*, and *T. onnurineus *species in which gene loss in one of the Pyrococcus species were detected.** The first worksheet contains protein functional annotations from the GenBank, arCOG, InterPro, PFam and KEGG Orthology databases. The second worksheet contains our manual functional annotations of 164 groups of orthologous genes used in the paper. The third worksheet contains numbers of clusters with genes annotated in HGT-DB as horizontally transferred. Click here for file

Additional file 2 **MS Excel file containing annotations of proteins of 911 orthologous clusters from the *P. furiosus*, *P. horikoshii*, *P. abyssi*, and *T. onnurineus *species containing one gene from each three Pyrococcus species and one gene from the *T. onnurineus *outgroup.** The first worksheet contains protein functional annotations from the GenBank, arCOG, InterPro, PFam and KEGG Orthology databases. The second worksheet contains our manual functional annotations of 911 groups of orthologous genes used in the paper. The third worksheet contains number of clusters with genes annotated in HGT-DB as horizontally transferred. Click here for file

Additional file 3**MS Excel file containing raw data: *d*_*R*_; *d*_*C*_; *Var*(*d*_*R*_); *Var*(*d*_*C*_); ; Γ_*i*_; *Var*(Γ_*i*_); Γ_max_; *Var*(Γ_max_). **Excel file contains four worksheet named according to the analyzed branches designated in Figure 2. Each of the four worksheets contains data on clusters in which positive selection was detected on the corresponding tree branch (Figure 2).Click here for file

Additional file 4 **MS Excel file containing functional annotations of proteins belonging to functional groups, whose genes are mainly subject to positive selection on the phylogenetic tree branches.** Worksheet contains protein functional annotations from the GenBank, arCOG, InterPro, PFam and KEGG Orthology databases. Click here for file

Additional file 5 **Adobe PDF file contains data on the relation between possible horizontal gene transfer events determined on the basis of the HGT-DB database data with positive selection events in gene clusters contained horizontally transferred genes. **Statistical significance of the relation between the events was estimated by the permutation test (see Methods). Click here for file

Additional file 6**MS Excel file containing raw data (; number of different types of codon changes; number of amino acid changes; total codon/amino acids number) for genes in sample I clusters (see Methods). **Excel file contains four worksheets named according to the analyzed tree branches given in Figure 2.Click here for file

Additional file 7 Gapless alignment of 16S rRNA gene sequences in FASTA format. Click here for file

Additional file 8 Adobe PDF file contains raw data on the GC content and codon mutations in 911 orthologous gene clusters. Click here for file
