# Supplementary material for: Molecular evolution of the hyperthermophilic archaea of the Pyrococcus genus: analysis of adaptation to different environmental conditions
Source: BMC Genomics. 2009 Dec 30;10:639. doi: 10.1186/1471-2164-10-639 (PMC2816203; doi:10.1186/1471-2164-10-639)
Supplement: Additional file 5 — Adobe PDF file contains data on the relation between possible horizontal gene transfer events determined on the basis of the HGT-DB database data with positive selection events in gene clusters contained horizontally transferred genes. Statistical significance of the relation between the events was estimated by the permutation test (see Methods). [file 1471-2164-10-639-S5.RTF]

Horisontal gene transfer versus regime of gene molecular evolution
Function group	Number of all clusters in group	Number of neutrally evolved and negative selected clusters with horisontal gene transfer 	Number of all positively selected clusters with horisontal gene transfer on branches*	Number of unique positively selected clusters with horisontal gene transfer on branches*	Number of all clusters with horisontal gene transfer in group	
			a	b	c	d	a	b	c	d		
INFORMATION STORAGE AND PROCESSING	
Translation; ribosomal structure and biogenesis	168	10	4	4	5	3	2	1	3	2	26	
Transcription	62	3	1	1	0	3	0	1	0	2	8	
Replication; recombination and repair	51	1	3	1	0	3	3	1	0	3	8	
CELLULAR PROCESSES AND SIGNALING	
Cell cycle control; cell division; chromosome partitioning	13	0	0	0	0	0	0	0	0	0	0	
Defense mechanisms	12	0	0	0	0	0	0	0	0	0	0	
Signal transduction mechanisms	10	0	0	0	0	0	0	0	0	0	0	
Cell wall/membrane/envelope biogenesis	19	0	0	0	1	0	0	0	1	0	1	
Cell motility	11	0	0	2	0	0	0	2	0	0	2	
Intracellular trafficking; secretion; and vesicular transport	11	1	0	1	0	1	0	0	0	0	3	
Posttranslational modification; protein turnover; chaperones	32	2	1	0	0	0	1	0	0	0	3	
METABOLISM	
Energy production and conversion	78	2	1	2	1	2	0	1	1	0	8	
Carbohydrate transport and metabolism	49	3	0	0	0	0	0	0	0	0	3	
Amino acid transport and metabolism	61	0	0	0	0	1	0	0	0	1	1	
Nucleotide transport and metabolism	38	0	1	0	0	1	1	0	0	1	2	
Coenzyme transport and metabolism	58	2	2	1	2	2	0	0	1	0	9	
Lipid transport and metabolism	15	0	0	0	0	0	0	0	0	0	0	
Inorganic ion transport and metabolism	46	1	0	0	0	0	0	0	0	0	1	
Secondary metabolites biosynthesis; transport and catabolism	7	0	0	0	0	0	0	0	0	0	0	
FUNCTION UNKNOWN	170	10	3	2	3	4	3	0	2	3	22	
Total	911	35	16	14	12	20	10	6	8	12	97	
* - Bold denote cases with excess of clusters with horizontal gene transfer (p≤0.05). Branch designations are as in Figure 2
