# Supplementary material for: Molecular evolution of the hyperthermophilic archaea of the Pyrococcus genus: analysis of adaptation to different environmental conditions
Source: BMC Genomics. 2009 Dec 30;10:639. doi: 10.1186/1471-2164-10-639 (PMC2816203; doi:10.1186/1471-2164-10-639)
Supplement: Additional file 8 — Adobe PDF file contains raw data on the GC content and codon mutations in 911 orthologous gene clusters. [file 1471-2164-10-639-S8.PDF]

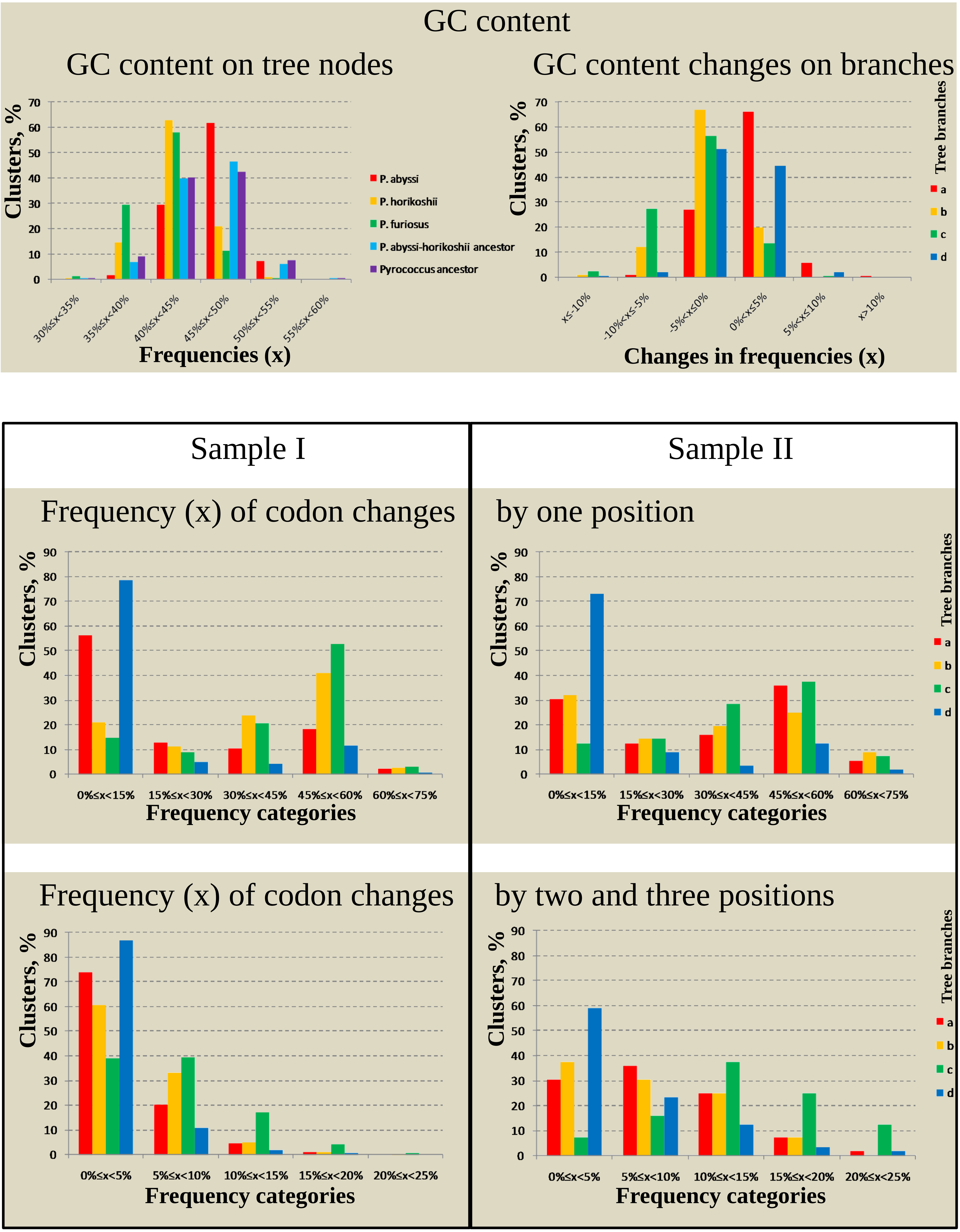
GC content
GC content on tree nodes
Clusters, %
0
10
20
30
40
50
60
70
30%≤x<35%
35%≤x<40%
40%≤x<45%
45%≤x<50%
50%≤x<55%
55%≤x<60%
Frequencies (x)
P. abyssi
P. horikoshii
P. furiosus
P. abyssi-horikoshii ancestor
Pyrococcus ancestor
GC content changes on branches
x≤-10%
-10%<x≤-5%
-5%<x≤0%
0%<x≤5%
5%<x≤10%
x>10%
Changes in frequencies (x)
Tree branches
a
b
c
d
Sample I
Sample II
Frequency (x) of codon changes
by one position
0%≤x<15%
15%≤x<30%
30%≤x<45%
45%≤x<60%
60%≤x<75%
Frequency categories
80
90
Frequency (x) of codon changes
by two and three positions
0%≤x<5%
5%≤x<10%
10%≤x<15%
15%≤x<20%
20%≤x<25%
Frequency categories
